# Consolidation radiographic morphology can be an indicator of the pathological basis and prognosis of partially solid nodules

**DOI:** 10.1186/s12890-022-02165-x

**Published:** 2022-09-28

**Authors:** Mei Xie, Jie Gao, Xidong Ma, Chongchong Wu, Xuelei Zang, Yuanyong Wang, Hui Deng, Jie Yao, Tingting Sun, Zhaofeng Yu, Sanhong Liu, Guanglei Zhuang, Xinying Xue, Jianlin Wu, Jianxin Wang

**Affiliations:** 1 Department of Respiratory and Critical Care, Chinese PLA General Hospital, the First Medical Centre, Beijing, 100835, People’s Republic of China; 2 Department of Radiology, Affiliated Zhongshan Hospital of Dalian University, Dalian, 116001 People’s Republic of China; 3 Department of Pathology, Chinese PLA General Hospital, Beijing, 100835 People’s Republic of China; 4 Department of Radiology, Chinese PLA General Hospital, Beijing, 100835 People’s Republic of China; 5 Center of Clinical Laboratory Medicine, First Medical Centre, Chinese PLA General Hospital, 100835 Beijing, People’s Republic of China; 6 Department of Thoracic Surgery, Tangdu Hospital of Air Force Military Medical University, Xi’an, 710038 Shanxi People’s Republic of China; 7 Department of Respiratory and Critical Care, Beijing Shijitan Hospital, Capital Medical University, 100038 Beijing, People’s Republic of China; 8 School of Medicine, Peking University, Beijing, 100871 People’s Republic of China; 9 Institute of Interdisciplinary Integrative Medicine Research, Shanghai University of Traditional Chinese Medicine, Shanghai, 201203 People’s Republic of China; 10 Shanghai Key Laboratory of Gynecologic Oncology, Renji Hospital, School of Medicine, Shanghai Jiao Tong University, 200000 Shanghai, People’s Republic of China

**Keywords:** Consolidation, Lung adenocarcinoma, Morphology, Part-solid nodule, Pathology, Tertiary lymphoid structure

## Abstract

**Background:**

Part-solid nodules (PSNs) have gradually shifted to defining special clinical subtypes. Commonly, the solid portions of PSNs show various radiological morphologies, of which the corresponding pathological basis and prognosis are unclear. We conducted a radiological–pathological evaluation to determine the histopathologic basis of different consolidation radiographic morphologies related to prognosis.

**Materials and methods:**

A cohort of 275 patients with a surgical pathological diagnosis of lung adenocarcinoma were enrolled. Preoperative computed tomography (CT) images of the PSNs were recorded and assessed. A panel of 103 patients with complete pathological specimens was selected to examine the radiological–pathological associations, and follow-up was performed to identify the prognosis.

**Results:**

Of the 275 patients, punctate consolidation was observed radiologically in 43/275 (15.7%), stripe consolidation in 68/275 (24.7%), and irregular consolidation in 164/275 (59.6%) patients. The radiological morphology of the solid components was significantly associated with the histopathological subtypes (*P* < 0.001). Visual punctate solid components on CT correlated with tertiary lymphoid structures, stripe solid components on CT correlated with fibrotic scar, and irregular solid components on CT correlated with invasion. PSNs with regular consolidation had a better prognosis than those with irregular consolidation.

**Conclusion:**

Radiological morphology of solid components in PSNs can indicate the pathological basis and is valuable for prognosis. In particular, irregular solid components in PSNs usually indicate serious invasive growth, which should be taken with caution during assessment.

## Background

The universal application of low-dose computed tomography (CT) in lung cancer screening has increased the detection rate of subsolid nodules. Persistent subsolid nodules are always considered malignant and have drawn considerable attention [1–3]. Based on the absence of solid components, subsolid nodules are classified as either pure ground-glass nodules (pGGNs) or part-solid nodules (PSNs) [4, 5]. Previous studies have asserted that subsolid nodules pathologically follow the developmental progress from preinvasive lesions to invasive adenocarcinoma. Correspondingly, a change from pGGNs to PSNs accompanied by focal solid component occurrence and obscuration of the internal pulmonary structure has been observed on CT images [6, 7].

Generally, the solid component size indicates the aggressive behavior of the disease and is correlated with patient prognosis [8–10]. Particularly, the size of solid portion was introduced to assign T categories in the eighth edition of the tumor-node-metastasis classification [11, 12]. However, it was recently proposed that the consolidation-to-tumor ratio or the size of the solid portion could not accurately predict pathological invasiveness and prognosis [13, 14]. Therefore, it is necessary to further investigate lung adenocarcinomas presenting as PSNs. Generally, the solid portions of PSNs show various morphologies. In our study, the solid components were categorized into three types, the corresponding pathological basis of which has not been reported before. Based on this background, we conducted a radiological–pathological evaluation to determine the histopathological basis of different consolidation radiographic morphologies related to prognosis.

## Materials and methods

### Patient cohorts

**Fig. 1:**
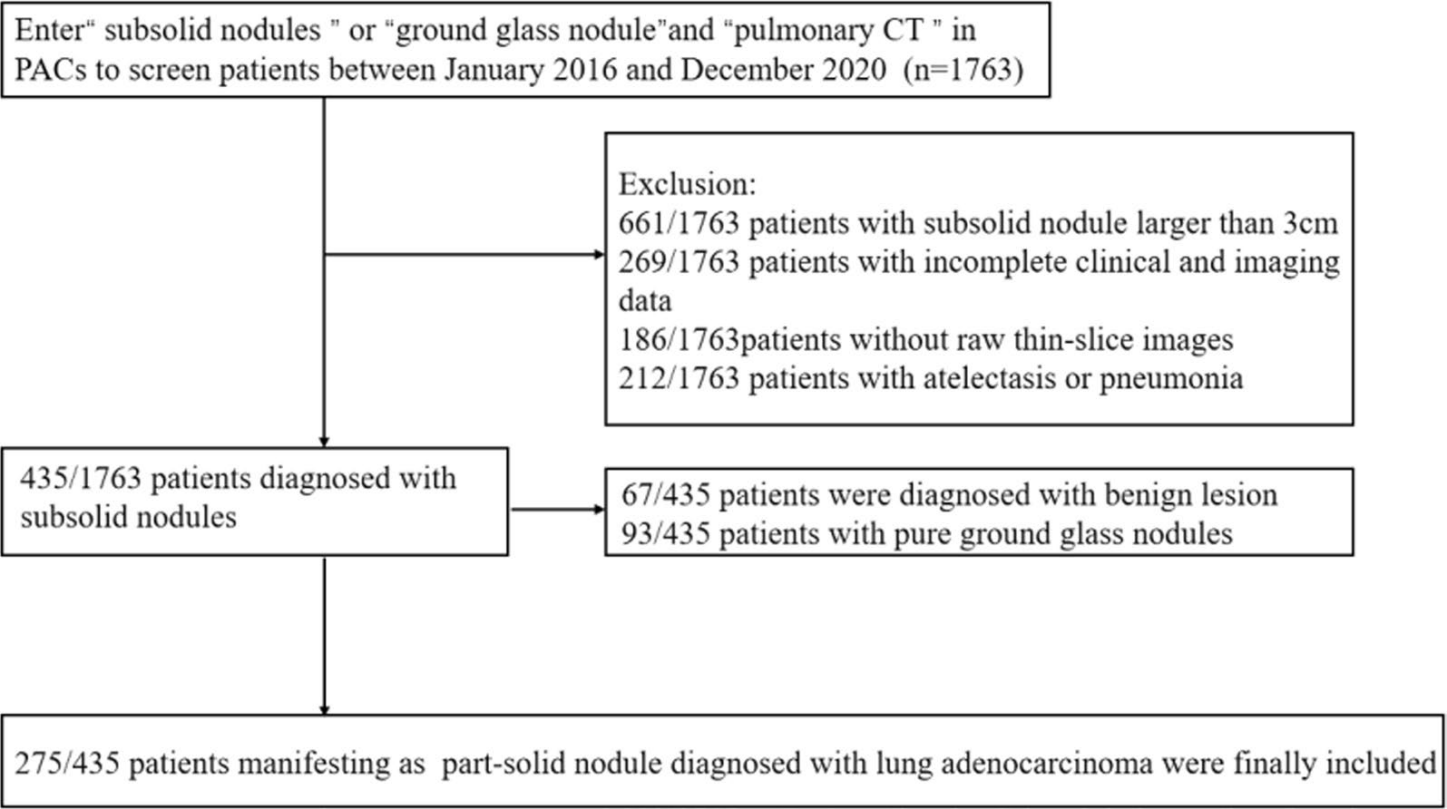
The exclusion and inclusion flowchart shows the number of patients

This retrospective study was approved by our institutional review board. We enrolled patients diagnosed with lung adenocarcinoma pathologically from January 2016 to January 2020 at the following hospitals: the General Hospital of the People’s Liberation Army, Affiliated Beijing Shijitan Hospital of Capital Medical University, and Affiliated Hospital of Qingdao University. The inclusion criteria were as follows: (a) patients with primary lung adenocarcinoma confirmed by postoperative pathology, (b) patients undergoing at least one CT examination within 1 month before surgery and with available complete thin-slice images, and (c) patients with a maximal diameter of subsolid nodule ≤ 3 cm. The exclusion criteria were as follows: (a) patients with incomplete clinical and CT data or without raw thin-slice images, (b) patients with a history of radiotherapy or chemotherapy before surgery, and (c) patients with pneumonia or atelectasis. Finally, 275 patients were included in this study (Fig. 1). We focused on the prognosis of 103 patients using pathological specimens. The follow-up data started on the surgical day, and any meaningful information, such as recurrence, was recorded.

### Computed tomography examinations and assessment

Chest CT examinations were performed using either a 64-Slice GE LightSpeed CT scanner (GE Healthcare, Beijing, China) or Siemens SOMATOM Sensation 128-Slice CT scanner (Siemens, Forchheim, Germany). The parameters for CT screenings were as follows: routine section thickness, 1.0–1.5 mm; section thickness after reconstruction, 0.625–1.25 mm; 80–120 kV; and 200–300 mAs.

**Fig. 2:**
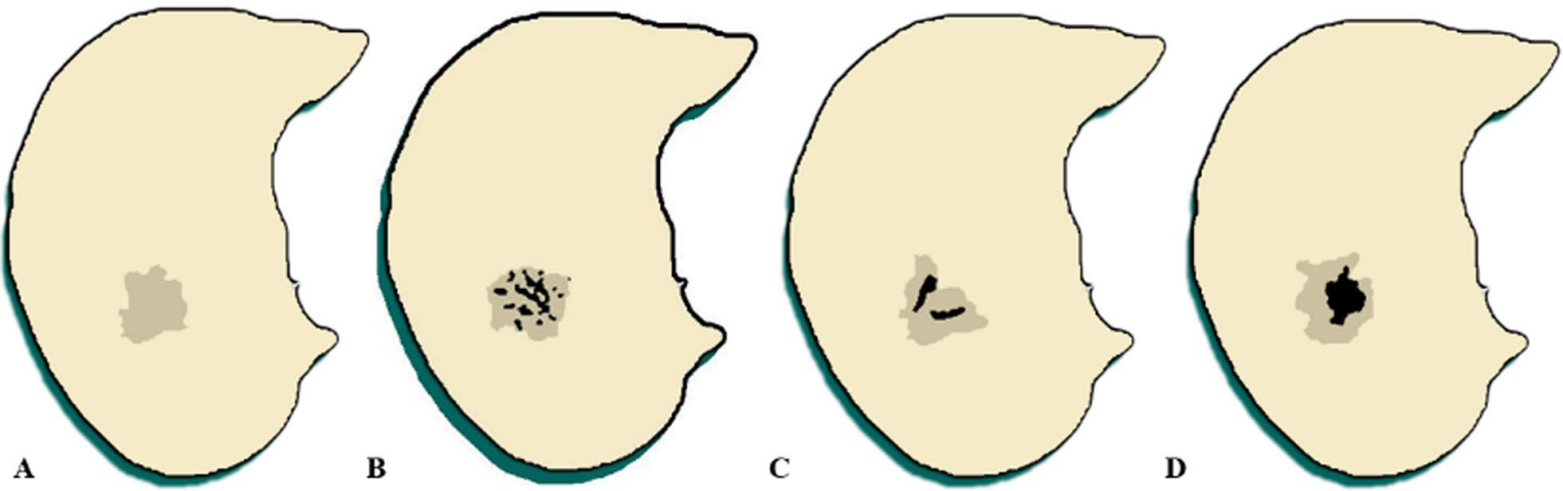
**a**–**d** Manifestation of subsolid nodule on axial computed tomography scans. **a** Pure ground-glass nodules with uniform density distribution; **b** part solid with punctate consolidation; **c** part solid with stripe consolidation; **d** part solid with irregular consolidation

These CT images were reviewed and assessed by setting the lung (width, 1500 HU; level, − 600 HU) and mediastinal (width, 350 HU; level, 40 HU) windows. A cardiopulmonary radiologist (C.W., with 20 years of experience) and a pulmonary radiologist (M.X., with 5 years of experience) conducted the evaluation work in consensus. The evaluation results were assessed and confirmed by a senior radiologist (J.W., with more than 24 years of experience). The following CT characteristics were recorded for each lesion: (a) lesion location, (b) lesion size (the average value of the maximum and minimum diameters on axial images), (c) solid component morphology findings (punctate, stripe, irregular), and (d) size (> 5 mm or < 5 mm). Figure 2 illustrates a schematic diagram of the morphological manifestation of consolidation in subsolid nodules.

### Pathological evaluation

All postoperative histopathological slides after staining with hematoxylin and eosin were analyzed in consensus by a senior pulmonary pathologist (J.G., with 20 years of experience) and a pulmonary pathologist (F.R., with 7 years of experience). The histopathological subtype was determined according to the international multidisciplinary classification of lung adenocarcinoma. Other recorded features included the presence of tertiary lymphoid structures, fibrotic scars within the tumors, and the presence of invasion.

### Statistical analyses

The Statistical Package for the Social Sciences version 26.0 (IBM Statistics, Armonk, NY, USA) was used for statistical data analyses. Continuous variables, such as age, tumor size, and smoking index, are expressed as means ± standard deviations with ranges. Categorical variables, including sex, smoking history, symptoms, and CT characteristics, are expressed as counts and percentages and compared using the Pearson chi-squared or Fisher exact test, when appropriate. The log-rank test and Kaplan–Meier analyses were used to estimate the survival curves of recurrence-free probability. *P*-values < 0.05 were considered statistically significant.

## Results

### Clinical and radiological characteristics

**Fig. 3:**
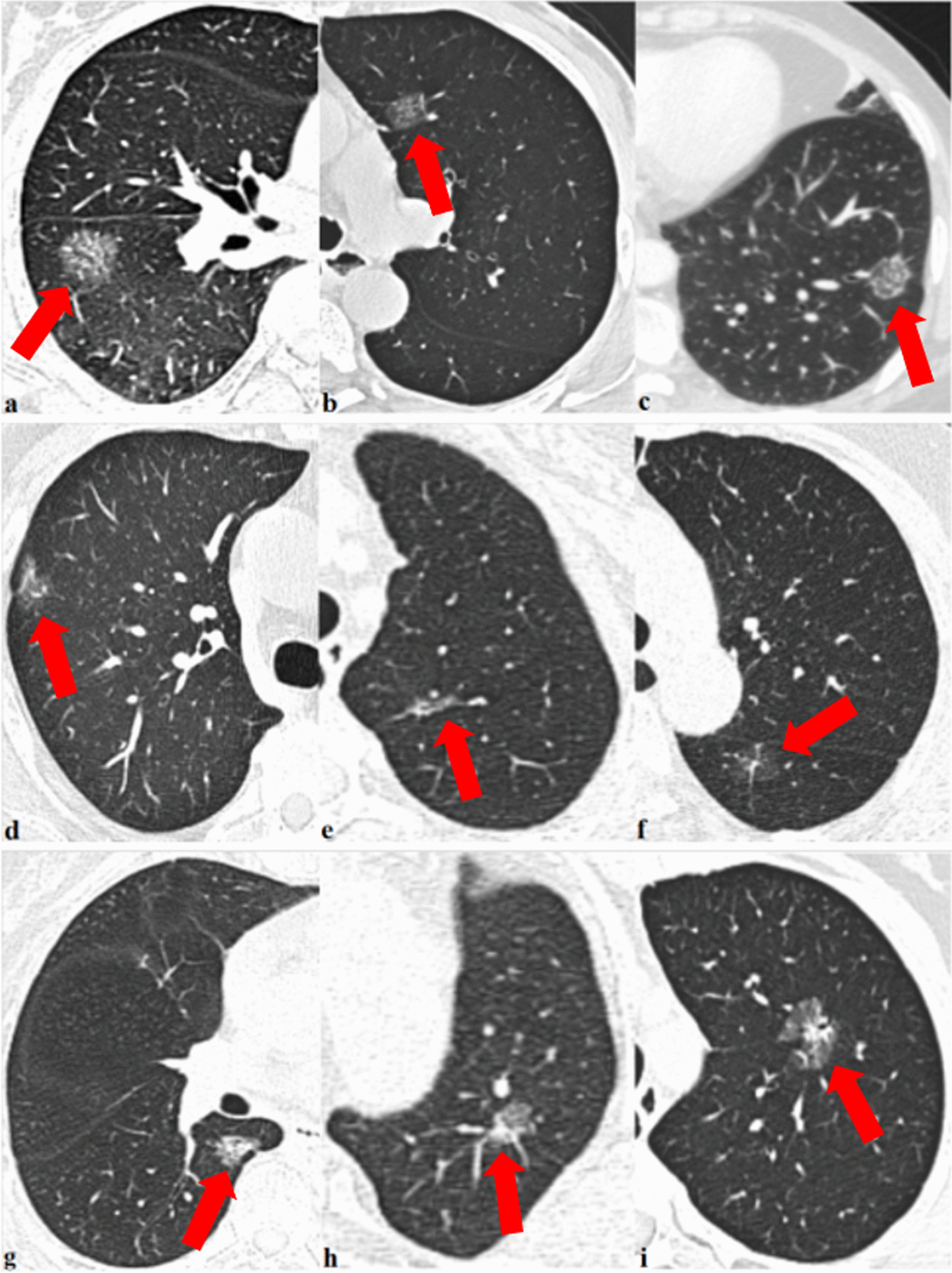
Axial computed tomography images show examples of solid component morphology, including the following: punctate consolidations smaller than 5 mm in part-solid nodules (**a**–**c**); stripe consolidations larger than 5 mm in part-solid nodules (**d**–**f**); irregular consolidations larger than 5 mm in part-solid nodules (**g**–**i**)

As demonstrated in Table 1, a cohort of 275 patients [mean age, 56.16 ± 10.31 (range, 27–80) years] with lung adenocarcinoma presenting with PSNs were enrolled in this study, including 185 (67.3%) female and 90 (32.7%) male patients. The average lesion size was 1.46 ± 0.52 (range, 0.59–2.89) cm. The radiographic morphology of consolidation was categorized into three types: punctate, stripe, or irregular (Fig. 3). Punctate, stripe, and irregular consolidations were observed radiologically in 43/275 (15.7%), 68/275 (24.7%), and 164/275 (59.6%) patients, respectively.

**Table 1:** Clinical and radiological characteristics of patients with lung adenocarcinoma presented as a subsolid nodule

Characteristics	Number	Datum
No. of patients	275	
Age (years)	–	56.16 ± 10.31
Sex	–	
Female	185	67.3%
Male	90	32.7%
Location	–	
Right upper lobe	110	40.0%
Right middle lobe	25	9.1%
Right lower lobe	40	14.5%
Left upper lobe	74	26.9%
Left lower lobe	26	9.5%
Size (cm)	–	1.46 ± 0.52
Morphology of solid components	–	
Punctate	43	15.7%
Stripe	68	24.7%
Irregular	164	59.6%
Size of solid components	–	
≤ 5 mm	122	44.4%
> 5 mm	153	55.6%
Recurrence	–	
Punctate	0	0
Stripe	1	0.9%
Irregular	5	4.9%

Datum is presented as mean ± standard deviations or percentages

*PSN  *Part-solid nodule

### Pathological basis

Additionally, 103 (37.5%) of the 275 patients had complete and available pathological specimens. The slides were then re-reviewed and evaluated. Punctate, stripe, and irregular consolidations were observed radiologically in 9/103 (8.7%), 28/103 (27.2%), and 66/103 (64.1%) patients, respectively. Pathological specimens included minimally invasive adenocarcinoma, invasive adenocarcinoma (lepidic predominant), invasive adenocarcinoma (acinar predominant), invasive adenocarcinoma (micropapillary predominant), invasive adenocarcinoma (papillary predominant), and invasive adenocarcinoma (solid predominant) in 8/103 (7.8%), 32/103 (31.0%), 52/103 (50.5%), 4/103 (3.9%), 4/103 (3.9%), and 3/103 (2.9%) patients, respectively. The visual radiological morphology of the solid components was significantly associated with histopathologic subtypes (*P* < 0.001) (Table 2).

**Table 2:** Correlation between radiographic morphology of consolidation and histological subtype

	Histopathological subtype				
	MIA	IVA-L	IVA-A	IVA-MP/P/S	*P*-value
Radiographic morphology					< 0.001
Punctate	5	3	1	0	
Stripe	2	16	10	0	
Irregular	1	13	41	11	

*MIA* Minimally invasive adenocarcinoma; *IVA-L* Invasive adenocarcinoma (lepidic predominant); *IVA-A* Invasive adenocarcinoma (acinar predominant); *IVA-MP/P/S* Invasive adenocarcinoma (micropapillary/papillary/solid predominant)

**Fig. 4:**
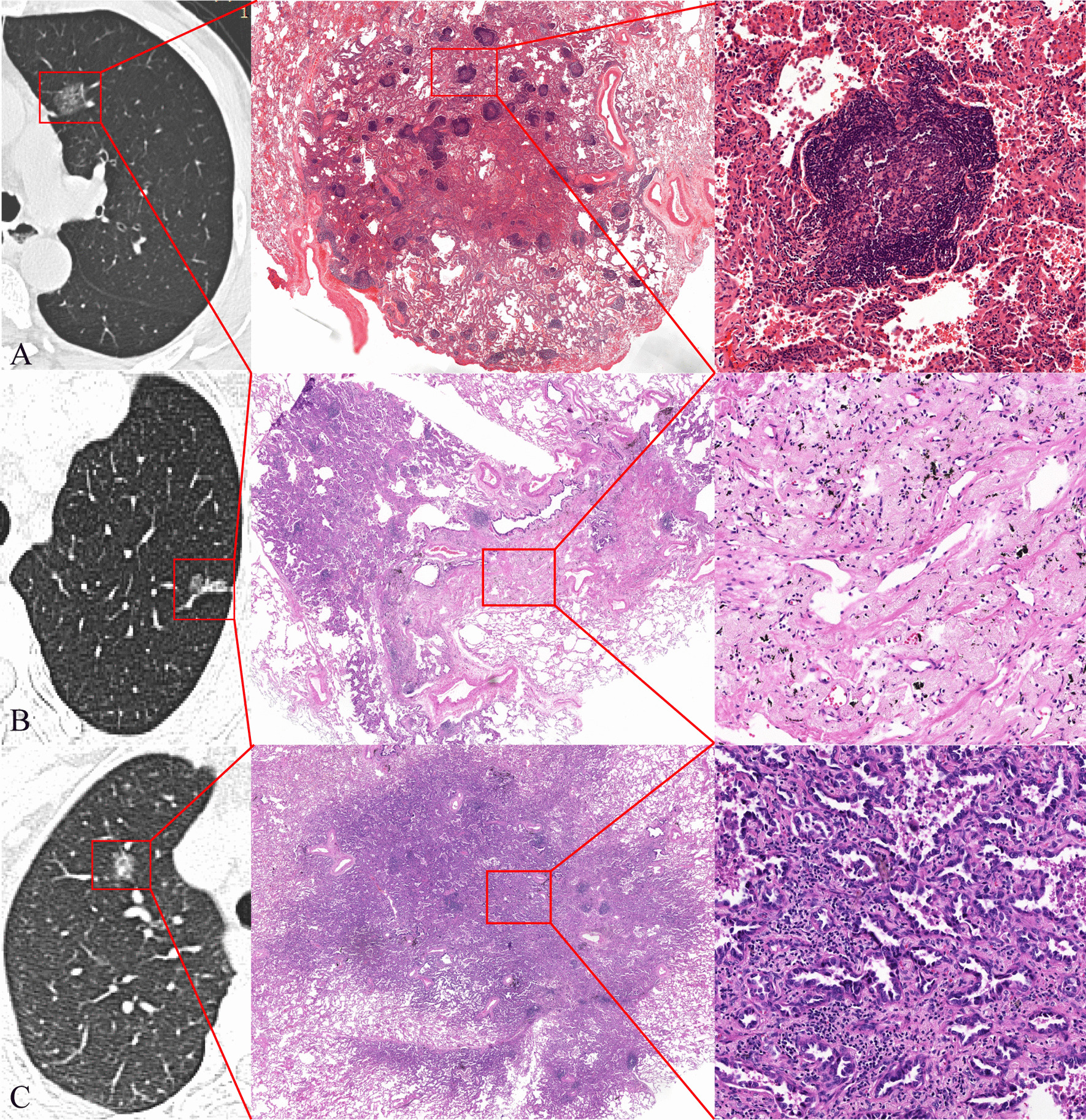
**A**–**C** Transverse axial computed tomography and corresponding pathological basis: **A** The punctate consolidation in part-solid nodule at the left upper lobe corresponds to tertiary lymphoid structure with a germinal center (red box); **B** The stripe consolidation in part-solid nodule at the left upper lobe corresponds to fibrotic scar formation without inner tumor cell or invasion observed (red box); **C** The irregular consolidation in part-solid nodule at the right upper lobe corresponds to invasion with inner acinar structure and lymphocyte clusters observed (red box)

We then retrospectively evaluated the correlation between radiological morphology presentations in 103 PSNs and their pathological findings. Tertiary lymphoid structures were clusters of lymphocyte aggregation in the tumor bed, and histopathological “invasion” was defined as tumor cells arranged in acinar, papillary, micropapillary, solid patterns or tumor cell-destroyed stroma and vascular structure. The results showed that visually punctate solid components on CT correlated with tertiary lymphoid structures in seven out of the nine (77.8%) patients (Fig. 4A), stripe solid components on CT correlated with fibrotic scarring in 17 out of the 28 (60.7%) patients (Fig. 4B), and irregular solid components on CT correlated with invasion in 41 out of the 66 (62.1%) patients (Fig. 4C). The radiological morphology of consolidation was also significantly associated with pathological basis (*P* < 0.001) (Table 3).

**Table 3:** Correlation between radiographic morphology of consolidation and pathological basis

	Pathological basis			
	Tertiary lymphoid structures	Fibrotic scarring	Invasion	*P*-value
Radiographic morphology				< 0.001
Punctate	7	1	1	
Stripe	1	17	10	
Irregular	5	20	41	

### Follow-up reports

**Fig. 5:**
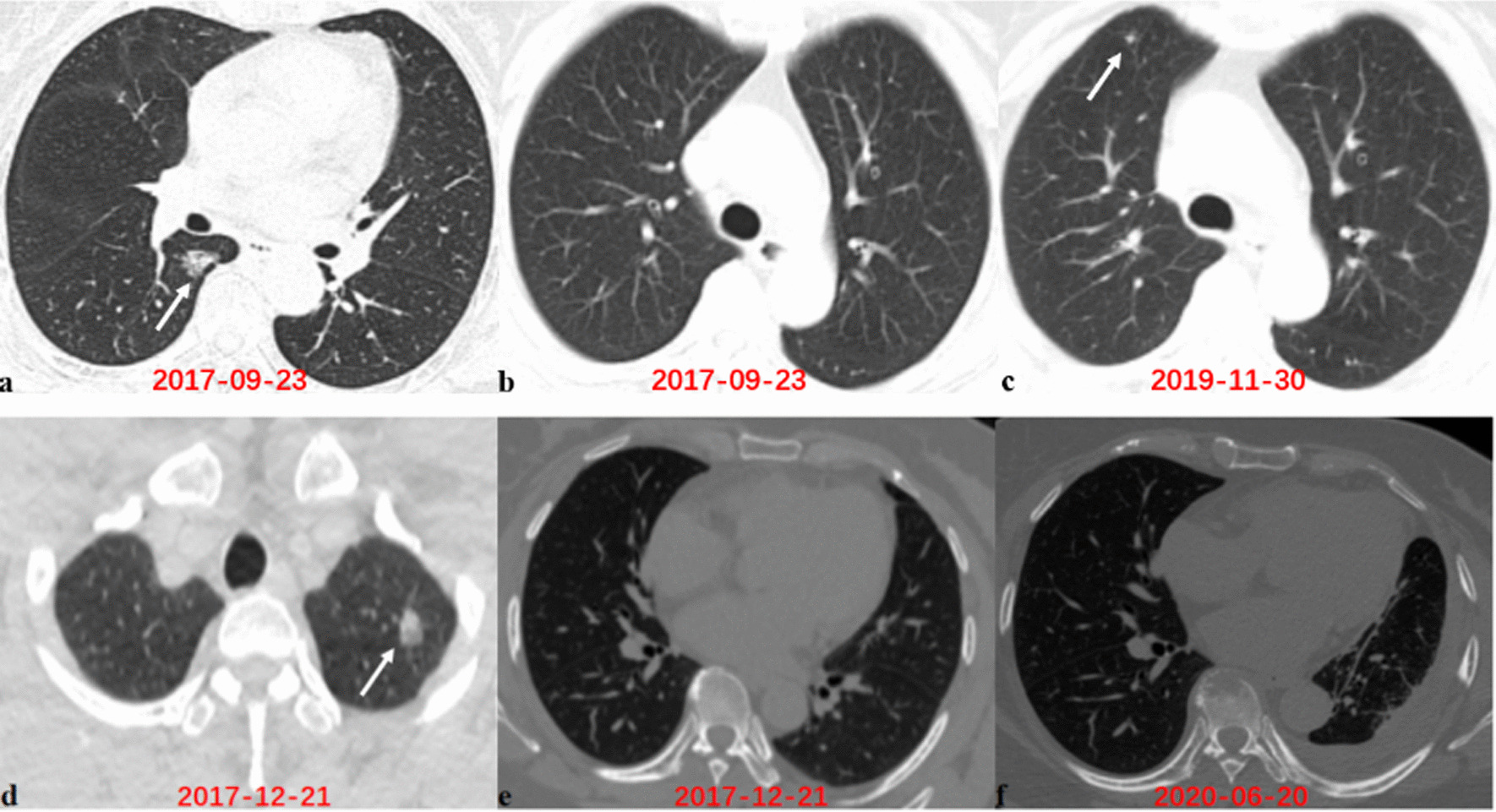
Axial computed tomography images show recurrence during follow-up. First row: A 62 year-old woman with lung adenocarcinoma. **a** The primary original lesion at the right lower lobe appears as a part-solid nodule with irregular consolidation (white arrow). **b**–**c** An emerging nodule diagnosed as metastasis at the right upper lobe (white arrow). Second row: A 55 year-old woman with lung adenocarcinoma (white arrow). **d** The primary original lesion at the left upper lobe appears as a part-solid nodule with irregular consolidation. **e**–**f** Osteolytic destruction of the vertebra and pleural thickening accompanied with pleural effusion both diagnosed as metastasis

**Fig. 6:**
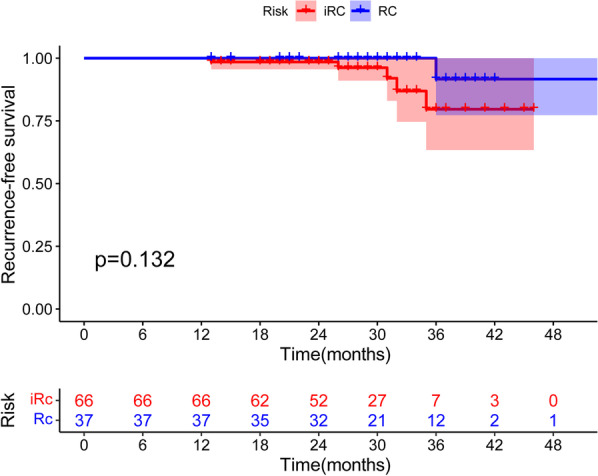
The Kaplan–Meier curve of recurrence-free survival outcomes in part-solid nodules with regular (punctate and stripe) morphology of consolidation (blue) and irregular morphology of consolidation (red)

In our study, follow-up was performed on 103 patients with complete pathological specimens. Recurrence was identified in 6 (5.8%) of the 103 patients. Of the six patients with recurrence, one had PSN with stripe consolidation and five had PSNs with irregular consolidation. As in the examples provided in Fig. 5a–c, a 62 year-old woman was diagnosed with lung adenocarcinoma, and the primary lesion appeared as a PSN with irregular consolidation located in the right lower lobe. Approximately 2 years after surgery, an emerging nodule diagnosed as a metastasis occurred in the upper lobe of the right lung. Another 55 year-old woman had a PSN with irregular consolidation in the left upper lobe and was diagnosed with lung adenocarcinoma. However, approximately 1 year after surgery, the patient experienced lung cancer metastasis. Osteolytic destruction of the vertebra and pleural thickening accompanied by pleural effusion were observed on her CT scans, as show in Fig. 5d–f. Additionally, all 103 patients were classified into two groups, regular (punctate and stripe) consolidation and irregular consolidation groups, according to the radiographic morphology of the solid components. The Kaplan–Meier survival curve illustrated that PSNs with a regular morphology of consolidation were associated with longer RFS than PSNs with an irregular morphology of consolidation. However, the difference was not statistically significant (*P* = 0.132), possibly because of the sample size, as shown in Fig. 6.

## Discussion

Lung adenocarcinoma is the leading fatal subtype of non-small cell lung cancer and is more prevalent among young female patients [15–17]. A recent study from China showed that 95.5% of lung adenocarcinomas detected by CT screening showed subsolid nodules [18]. In this study, we categorized the consolidation of subsolid nodules into dots, stripes, or irregularities and found that the radiological morphology of solid components was correlated with dissimilar pathological basis and prognosis.

The punctate consolidation in PSN on CT scans was previously considered to be a tiny invasion focus [19]. However, it was first noted in our study that punctate consolidation may be a pathological tertiary lymphoid structure. Tertiary lymphoid structures are clusters of lymphocyte aggregates in non-lymphatic tissues [20, 21] and have been shown to participate in the antitumor immune process. Patients with tertiary lymphoid structures have been reported to benefit from long-term survival [22–24]. The solid component of the striae on CT correlated with the presence of fibrotic scars pathologically. Fibrotic scar formation has been proposed as a unique histopathological characteristic of lung adenocarcinoma and is common in the process of carcinogenesis [25, 26]. However, fibrotic scar formation was excluded from the definition of “invasion” and reported to be associated with prognosis [27, 28]. We hypothesized that fibrotic scar formation was a type of antitumor repair mechanism, and that no invasive components, such as tumor cells or vascular involvement, were observed within it. Notably, clinicians should be alert when they encounter irregular solid components of PSN on CT scans because the pathological basis is closely related to the extension of invasive foci, showing an increase in tumor invasiveness. In particular, part of the invasion is accompanied by fibrous scarring, which makes the tumor more contractile, and some CT features, such as the spicule sign, appear on CT scans [29, 30].

Follow-up results showed that no recurrence occurred in PSNs with punctate solid components. In contrast, PSN with irregular solid components experienced recurrence or metastasis. Previous studies have explored the correlation between the size or proportion of solid components of PSNs and prognosis in patients with lung adenocarcinoma. The conclusion was that the solid component size was not prognostic in part-solid lung cancer due to the presence of ground-glass opacity [31–33]. Ground-glass opacity with a minimally invasive nature is always favorable for the prognosis. Interestingly, we demonstrated a strong positive correlation between the radiological morphology of the solid component and the histopathological subtype. Histopathological categories were proposed based on evidence of patient survival. Therefore, estimations of the pathological basis and prognosis based on the radiological morphology of solid components are desirable. Based on the results of this study, we hypothesized that the existence of a tertiary lymphoid structure or fibrotic scar formation may be another explanation for the ideal long-term survival of PSN with solid components. Consequently, we propose that the radiological morphology of the solid component in PSNs is also meaningful for predicting the histopathological basis and prognosis of patients with lung adenocarcinoma.

Our study has some limitations. First, this was a retrospective study with a small sample size, and further studies from multiple centers are required to confirm the results. Second, deviation in the accurate judgment of CT signs might exist because of the small nodules. Third, there was no precise definition of the three different solid component morphologies in PSN, and further refinement is required in future studies. Finally, due to the indolent nature of subsolid nodules, the follow-up time should be sufficiently long and require further confirmation.

## Conclusion

In conclusion, the radiological morphology of the solid components in PSN can reflect a pathological basis and is valuable for prognosis. In particular, irregular solid components in PSNs usually indicate serious aggressive behavior, which should be taken with caution during assessment.

## Data Availability

The datasets used in this study are available from the corresponding author upon reasonable request.

## Abbreviations

CT: Computed tomography
pGGNs: Pure ground-glass nodules
PSNs: Part-solid nodules
